# Changes of Spontaneous Oscillatory Activity to Tonic Heat Pain

**DOI:** 10.1371/journal.pone.0091052

**Published:** 2014-03-06

**Authors:** Weiwei Peng, Li Hu, Zhiguo Zhang, Yong Hu

**Affiliations:** 1 Department of Orthopaedics and Traumatology, The University of Hong Kong, Pokfulam, Hong Kong; 2 Key Laboratory of Cognition and Personality (Ministry of Education) and School of Psychology, Southwest University, Chongqing, China; 3 Department of Electrical and Electronic Engineering, The University of Hong Kong, Pokfulam, Hong Kong; Institute of Psychology, Chinese Academy of Sciences, China

## Abstract

Transient painful stimuli could induce suppression of alpha oscillatory activities and enhancement of gamma oscillatory activities that also could be greatly modulated by attention. Here, we attempted to characterize changes in cortical activities during tonic heat pain perception and investigated the influence of directed/distracted attention on these responses. We collected 5-minute long continuous Electroencephalography (EEG) data from 38 healthy volunteers during four conditions presented in a counterbalanced order: (A) resting condition; (B) innoxious-distracted condition; (C) noxious-distracted condition; (D) noxious-attended condition. The effects of tonic heat pain stimulation and selective attention on oscillatory activities were investigated by comparing the EEG power spectra among the four experimental conditions and assessing the relationship between spectral power difference and subjective pain intensity. The change of oscillatory activities in condition D was characterized by stable and persistent decrease of alpha oscillation power over contralateral-central electrodes and widespread increase of gamma oscillation power, which were even significantly correlated with subjective pain intensity. Since EEG responses in the alpha and gamma frequency band were affected by attention in different manners, they are likely related to different aspects of the multidimensional sensory experience of pain. The observed contralateral-central alpha suppression (conditions D vs. B and D vs. C) may reflect primarily a top-down cognitive process such as attention, while the widespread gamma enhancement (conditions D vs. A) may partly reflect tonic pain processing, representing the summary effects of bottom-up stimulus-related and top-down subject-driven cognitive processes.

## Introduction

Transient painful stimulation could induce remarkable changes on spontaneous oscillatory activity over a wide range of frequency bands, e.g., suppression of alpha band oscillations (8–14 Hz) over the contralateral sensorimotor cortex [1], [2], [3], [4], [5], and enhancement of gamma band oscillations (30–100 Hz) over the contralateral primary somatosensory cortex [6], [7], [8]. Both alpha oscillation suppression and gamma oscillation enhancement have been reported to be significantly correlated with the subjective pain intensity [2], [6], [8], [9]. Functionally, pain-induced suppression of alpha oscillations was related to cortical excitability, which facilitated the alerting function of pain leading to preferred processing [5], [10], and pain-induced enhancement of gamma oscillations represented cortical activity subserving pain perception, which constituted the mechanism for integrating low-level cortical processing of basic stimulus features and high-level cognitive processes (e.g., attention and anticipation) [8], [11].

The cortical responses induced by transient pain dominantly represented the immediate impact on cortical activities related to the onset of pain perception, which could not mimic chronic pain experience that was persistent, with or without pronounced fluctuations, over a period of time [12], [13], [14], [15]. Thus, tonic pain, induced by noxious stimulation with extended duration, was frequently adopted to explore neural mechanisms related to persistent pain experience instead of the onset of pain perception [12],[13],[14],[15],[16],[17],[18],[19]. However, electrophysiological studies reported inconsistent findings about the effect of tonic pain on spontaneous oscillatory activity [12], [13], [14], [16], [17], [18], [19], [20]. By comparing with tonic but non-painful stimulation condition, some studies reported suppression of alpha oscillations, induced by tonic pain, in frontal-central, temporal, or parieto-occipital regions [13], [14], [16], [17], [18], [20], while others described enhancement of alpha oscillations over frontal or parieto-occipital regions [12], [19]. Meanwhile, changes in gamma oscillations induced by tonic pain were rarely reported.

Another domain of tonic pain research that has not been explored is to study the cerebral mechanisms of attentional modulation on tonic pain processing, which could help to better understand the psychological factors in pain. In both basic and clinical contexts, attention towards pain can aggravate chronic pain and the associated subjective experience [21], [22], [23]. Similarly, distraction/attention could alter pain associated cortical responses (e.g., spontaneous oscillatory activity) [3], [11], [24], [25], [26], [27]. Those changes in spontaneous oscillatory activity may represent attentional augmentation of processing, since the suppression of alpha oscillations was more widespread and intense when directing attention to than being distracted from transient pain [3], and the enhancement of gamma oscillations over sensorimotor areas increased with attention directed to transient pain [11], [26], [27]. However, it is still not clear about how distraction/attention modulate the tonic pain induced changes of spontaneous oscillatory activity.

In the present study, we aimed to understand the function of oscillatory neural responses to tonic pain stimulation by (1) characterizing tonic pain induced changes in spontaneous oscillatory activity, and (2) investigating the influence of attention/distraction on the explored modulations of spontaneous oscillatory activity. Continuous electroencephalographic (EEG) data were collected from 38 healthy volunteers under four conditions (5-minute recordings for each condition): (A) resting condition, (B) innoxious-distracted condition, (C) noxious-distracted condition, and (D) noxious-attended condition. The EEG power spectra, including both alpha and gamma oscillations, among four experimental conditions were comprehensively compared. Also, the relationship between subjective pain intensity and tonic pain induced changes in spontaneous oscillatory activity was assessed. Then, with the obtained EEG oscillatory features that are related to tonic heat pain, the minimal length of EEG recording for distinguishment among different conditions was identified.

## Materials and Methods

### Subjects

Thirty-eight healthy right-handed volunteers (21 females) with a mean age of 21 years (range: 19–25 years) participated in the study. None reported acute or chronic pain at the time of examination. This study was approved by the local ethics committee (Institutional Review Board of the University of Hong Kong/Hospital Authority Hong Kong) and conducted in accordance with the principles of the Helsinki declaration. All subjects gave written informed consent before participation.

### Stimulus

A thermal contact-heat stimulator (PATHWAY sensory evaluation system, Medoc Ltd., Israel), with a thermode having a circular contact area of 572.5 mm^2^ (27 mm in diameter), was employed in the present study to deliver innoxious and noxious stimuli. For each stimulation condition, the rates of temperature increase and decrease were 70°C/s and 40°C/s, respectively. The thermode was slightly repositioned after each stimulation condition to minimize nociceptor sensitization or habituation.

### Experimental Design

**Figure 1:**
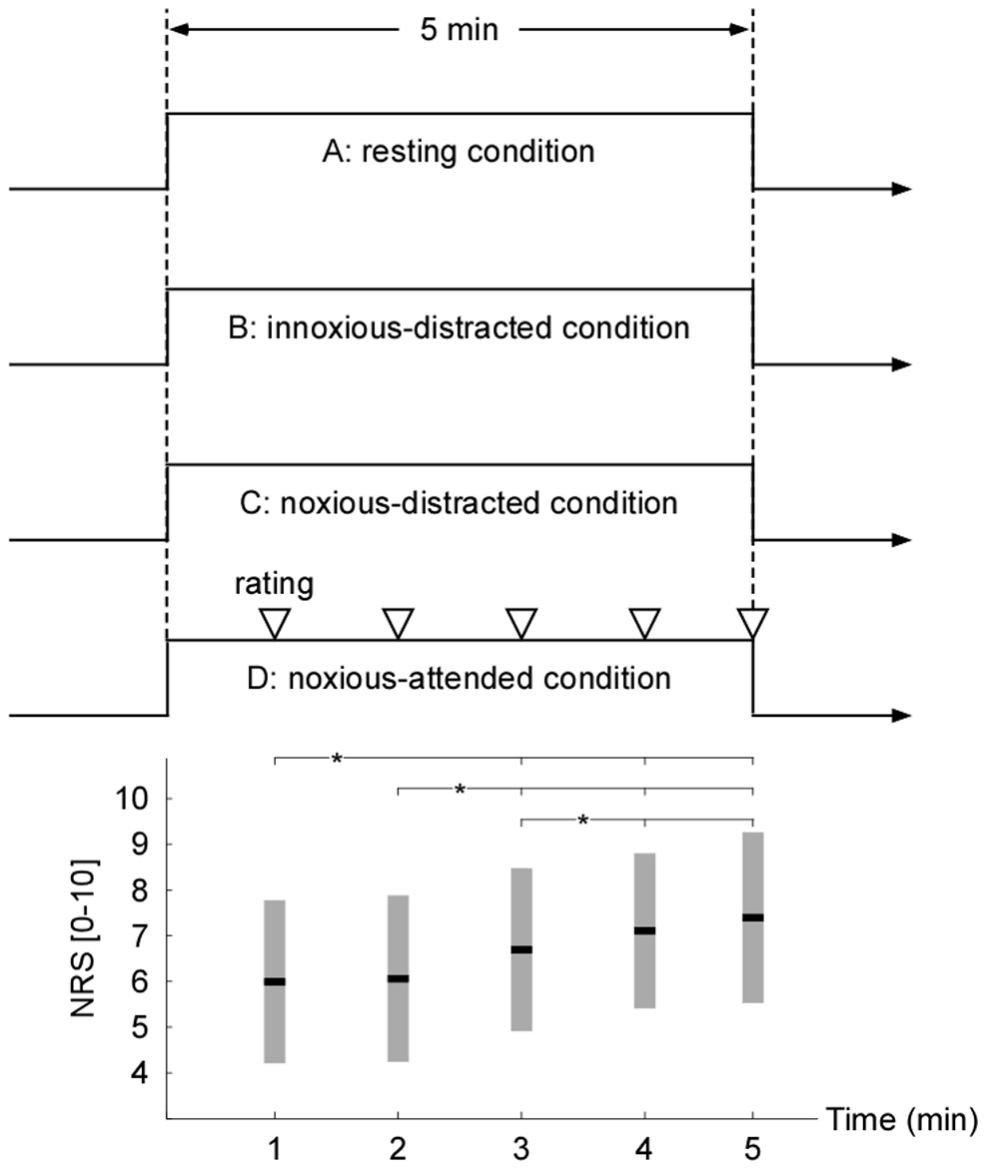
Experimental design and behavioral results. Top panel: The experiment consisted of four stimulation conditions (A: resting condition; B: innoxious-distracted condition; C: noxious-distracted condition; D: noxious-attended condition), which were presented in counterbalanced order within a single session. Each stimulation condition last for 5 minutes with a 10-minute break between consecutive stimulation conditions. Bottom panel: In condition D, the reported NRS scores at the end of each minute were compared to assess the possible change of pain perception with the prolonged duration of noxious stimulation. Error bars represent, for each minute, ± SD across subjects. Asterisk (*) indicates a significant difference (P<0.05, Tukey’s post hoc tests).

The subjects were seated on a comfortable chair in a silent, temperature-controlled room. Prior to data collection, subjects were acquainted with the pain rating scale and experimental procedures. At each stage of the study, the participants were reminded that they could withdraw from the experiment at any time for any reason, but none did so. During the experiment, four stimulation conditions (A: resting condition; B: innoxious-distracted condition; C: noxious-distracted condition; D: noxious-attended condition) were presented in a counterbalanced order within a single session (Fig. 1), and each stimulation condition last for 5 minutes with a 10-minute break between consecutive stimulation conditions.

In the resting condition (A), subjects were instructed to keep relaxed and eyes open, meanwhile, no stimulation was applied. In the innoxious-distracted condition (B), subjects were asked to count backwards every 3 s from a randomly chosen 4-digit number, and an innoxious stimulus (36°C) was continuously imposed on the non-dominant (left) volar forearm. The use of condition B attempted to control the possible pressure sensation associated with the contact between the thermode and the forearm, and to maintain level of vigilance throughout the 5-minute recording session. In the noxious-distracted condition (C), subjects were also asked to count backwards every 3 s from a randomly chosen 4-digit number, and noxious stimulus was continuously imposed on the non-dominant (left) volar forearm (the position was slightly different from condition B). Note that the noxious stimulus was delivered with a temperature evoking a pain experience with a numeric rating scale (NRS) score of 6 during the 30-s stimulation test (0: no sensation, 2: sensory threshold, 4: pain threshold, and 10: the worst imaginable pain) [28], [29], [30]. In the noxious-attended condition (D), subjects were instructed to pay attention to the continuously imposed noxious stimulus (NRS score = 6) that was imposed on the non-dominant (left) volar forearm (the position was slightly different from conditions B and C). At the end of each minute, subjects were asked to verbally rate the perceived intensity of pain perception on the 0–10 NRS.

It should be noted that since psychology state (e.g., attention) could modulate the perception of experimentally induced pain [21], [22], [23], it is highly likely that the subjects would have different perception to the tonic pain in conditions C (noxious-distracted) and D (noxious-attended), even the stimulus intensity was same for two conditions. In addition, even the temperatures of noxious stimuli were consistent in conditions C and D, the perceived pain intensities may vary at every moment in each condition. Such experiment design in the present study allowed us to identify the global effects of tonic heat pain on spontaneous oscillatory activity by comparing EEG activity between condition D and A, and disclose attention-related effects on spontaneous oscillatory activity by comparing EEG activity between conditions D and B, and between conditions D and C.

### Behavioral Data Analysis

In condition D, the reported NRS scores were compared using a 5-level (5 minutes) one-way repeated measures analysis of variance (ANOVA) with a statistical significance level of P<0.05, to assess possible changes of pain perception with the prolonged duration of the noxious stimulation. Mauchly’s test was applied to assess possible violations of sphericity [31]. If the assumption of sphericity was violated (P<0.05), the degrees of freedom were adjusted (ε <0.75: Greenhouse-Geisser correction, ε >0.75: Huynh and Feldt correction). When the main effect of the ANOVA was significant, Tukey’s post hoc tests were performed.

### EEG Recording

The EEG data were recorded using a 64-channel Brain Products system (pass band: 0.01–100 Hz, sampling rate: 1000 Hz) using a standard EEG cap based on the extended 10–20 system. The nose was used as the reference channel, and all channel impedances were kept lower than 10 kΩ. To monitor ocular movements and eye blinks, electro-oculographic signals were simultaneously recorded from four surface electrodes, one pair placed over the upper and lower eyelid, the other pair placed 1 cm lateral to the outer corner of the left and right orbit.

### EEG Data Analysis

#### Preprocessing

EEG data were preprocessed using EEGLAB [32], an open source toolbox running under the MATLAB environment. For each condition, continuous EEG data were band-pass filtered between 1 and 100 Hz. To rule out possible brain responses related to the sudden change of stimulation (i.e., the onset and offset of the stimulation), EEG data collected during the first and the last minutes were discarded, and the remaining EEG data from the second to the fourth minutes were segmented into 180 EEG epochs using a window analysis time of 1 s. EEG segments contaminated by strong muscle artifacts were manually rejected by visual inspection. Epochs contaminated by eye-blinks and movements were corrected using an independent component analysis algorithm [25], [33], [34]. In all datasets, independent components with a large EOG channel contribution and a frontal scalp distribution were removed. Furthermore, in condition D, epochs with speech artifacts, which were caused by verbally rating the perceived intensity of pain perception, were discarded from the following analysis.

#### EEG spectral analysis

For each subject and each stimulation condition, the segmented EEG epochs were transformed to the frequency domain using a discrete Fourier transform [35], yielding power spectra (in µV^2^) ranging from 1 to 100 Hz. For each electrode, the obtained single-epoch power spectra were averaged across epochs to enhance the signal-to-noise ratio. Since we were specifically interested in spectral power difference across conditions, the averaged power spectra were normalized across stimulation conditions (A, B, C, and D), and expressed as z values at each frequency point (subtracting the mean and dividing by the standard deviation of the spectra). Such z-score normalization method, which has been popularly applied in neuroimage studies [36], [37], [38], was quite useful to ensure (almost) equal contributions from each subject for the following comparisons of power spectra across the conditions. Considering condition A (resting condition) is important to be included as a baseline condition (the same in several previous studies [12], [13], [14], ), our experimental design actually did not contain two independent variables (i.e., attention and stimulus). If condition A is innoxious-attended condition, or if condition B is resting-distracted condition, it would be more proper to perform two-way repeated-measures ANOVA. Instead, we think it would be better to perform one-way repeated-measures ANOVA based on the current experimental design. Thus, a 4-level (conditions A, B, C, and D) point-by-point one-way repeated measures ANOVA was performed on the normalized power spectra to identify possible frequency intervals with significant difference among stimulation conditions. To account for multiple comparisons induced by different channels and frequency points, the significance level (P value) was corrected using a false discovery rate procedure [39].

The summarized spectral power within the alpha frequency band at contralateral-central electrodes (C2, C4, CP2, and CP4), and within the gamma frequency band at frontal-central (Fz, FC1, FC2, and Cz) and ipsilateral-central (C1, C3, CP1, and CP3) electrodes, were calculated for each subject and each stimulation condition, and then compared using a 4-level (4 stimulation conditions) one-way repeated measures ANOVA with a statistical significance level of P<0.05. When the main effect of the ANOVA was significant, Tukey’s post hoc tests were performed.

The relationship between averaged EEG responses (spectral power difference between the condition D and other three conditions [A, B, and C]) and subjective pain intensity (NRS scores in condition D) during the interval of 2^nd^ to 4^th^ minutes was assessed by performing linear correlation analysis for each electrode and each the frequency interval showing significant differences among stimulation conditions (i.e., alpha and gamma bands), which were identified from the preceding analysis. Within the spatial regions that showed the strongest correlation (contralateral-central electrodes [C2, C4, CP2, and CP4] for the alpha frequency band; prefrontal-central [AF3, AF4, F1, Fz, and F2] and ipsilateral-posterior [CP1, CP3, CP5, P1, P3, and P5] electrodes for the gamma frequency band), correlation coefficients and their significance levels were calculated for each minute (from the 2^nd^ to 4^th^ minute) to assess the time-varying relationship between tonic pain related oscillatory activities and subjective pain intensity.

To assess the time-varying spectral power distribution during tonic heat pain perception, spectral power were averaged from every 10 consecutive epochs (shifting from 2^nd^ to 4^th^ minutes [1–180 epochs] with 1s [1 epoch] in time step), yielding power spectral (in µV^2^) ranging from 1 to 100 Hz in frequency and from 2^nd^ to 4^th^ minute in latency. Then, the obtained spectral power densities were normalized across conditions (subtracting the mean and dividing by the standard deviation of the spectra), and expressed as z values at each time and frequency point. Note that spectral power density that may be contaminated by speech artifacts resulting from reporting NRS at the end of each min were eliminated. As we noted that tonic heat pain related oscillatory activity was contralateral central alpha oscillatory activity, and frontal and ipsilateral central gamma oscillatory activity, grand averaged time varying normalized spectral power difference (D–B, D–C) were measured at contralateral central electrodes (C2, C4, CP2, and CP4), and power differences (D–A) were measured at frontal central (Fz, FC1, FC2, and Cz) and ipsilateral central (C1, C3, CP1, and CP3) electrodes. Also, grand averaged time-varying curve of normalized alpha spectral power (10–15 Hz) at contralateral central electrodes (C2, C4, CP2, and CP4), and those of normalized gamma spectral power (30–100 Hz) at frontal (Fz, FC1, FC2, and Cz) and ipsilateral central (C1, C3, CP1, and CP3) electrodes were computed. A 4-level (conditions A, B, C, and D) point-by-point one-way repeated measures ANOVA was performed on the time-varying normalized power spectra to identify possible time intervals with significant difference among stimulation conditions.

Since the signal-to-noise ratio of brain responses elicited by tonic stimulation is markedly lower compared with transient stimulation (i.e., the onset or offset of stimulus), tonic pain induced EEG response would not be reliably detected using short-interval EEG data. EEG signals recorded in a sufficiently long duration (e.g., 3 minutes in the present study) would be necessary to achieve a possible significant differentiation among stimulation conditions. Indeed, it would be very interesting and instructive to explore the minimal length of EEG recordings that we need to achieve a significant distinguishment among different tonic stimulation conditions. Thus, the minimum EEG recording interval to significantly distinguish different stimulus conditions was assessed by measuring tonic heat pain related activities (identified in the previous steps). Firstly, single-epoch normalized power in the alpha and gamma bands were respectively calculated from their significant frequency intervals (i.e., alpha and gamma frequency bands) and dominant spatial regions (contralateral-central electrodes [C2, C4, CP2, and CP4] for the alpha frequency band; frontal-central [Fz, FC1, FC2, and Cz] and ipsilateral-central [C1, C3, CP1, and CP3] electrodes for the gamma frequency band). Then, spectral power in the alpha and gamma bands from consecutive epochs (number ranging from 5 to 70 in step of 5) were averaged and compared using 4-level (4 stimulation conditions) one-way repeated measures ANOVA. Note that such an analytical step was repeatedly performed by changing the combination of consecutive epochs (shifting from the beginning to the end of the whole 180 epochs). Lastly, for each length of consecutive epochs, the percentage of combinations with significant difference among stimulation conditions out of the total combinations was calculated. When the main effect of the ANOVA was significant, Tukey’s post hoc tests were performed.

## Results

### Behavioral Results

During the 30-s stimulation test, the average temperature (mean ± SD) that evoked a pain experience with NRS scores of 6 was 45.4±1.3°C. In condition D, the reported NRS scores at the end of each minute were 6.01±1.72, 6.03±1.77, 6.68±1.68, 7.12±1.61, and 7.38±1.80, respectively (Fig. 1). As revealed by 5-level one-way repeated measures ANOVA, the NRS scores were significantly different (F (2.02, 85.56) = 20.57, P<0.001, partial Eta squared = 0.35). Post hoc comparisons revealed that the NRS scores significantly increased with prolonged duration of the noxious stimulation (NRS_1_< NRS_3_; NRS_1_< NRS_4_; NRS_1_< NRS_5_; NRS_2_< NRS_3_; NRS_2_< NRS_4_; NRS_2_< NRS_5_; NRS_3_< NRS_4_; NRS_3_< NRS_5_; P<0.01 for all comparisons [NRS_n_: the reported NRS scores at the end of the n-th minute in condition D]). These results indicated the longer the noxious stimulation, the stronger the intensity of pain perception, especially from the second to the fourth minute, which demonstrated that no obvious perceptive habituation to the noxious stimulation existed in the present data.

### Electrophysiological Results

**Figure 2:**
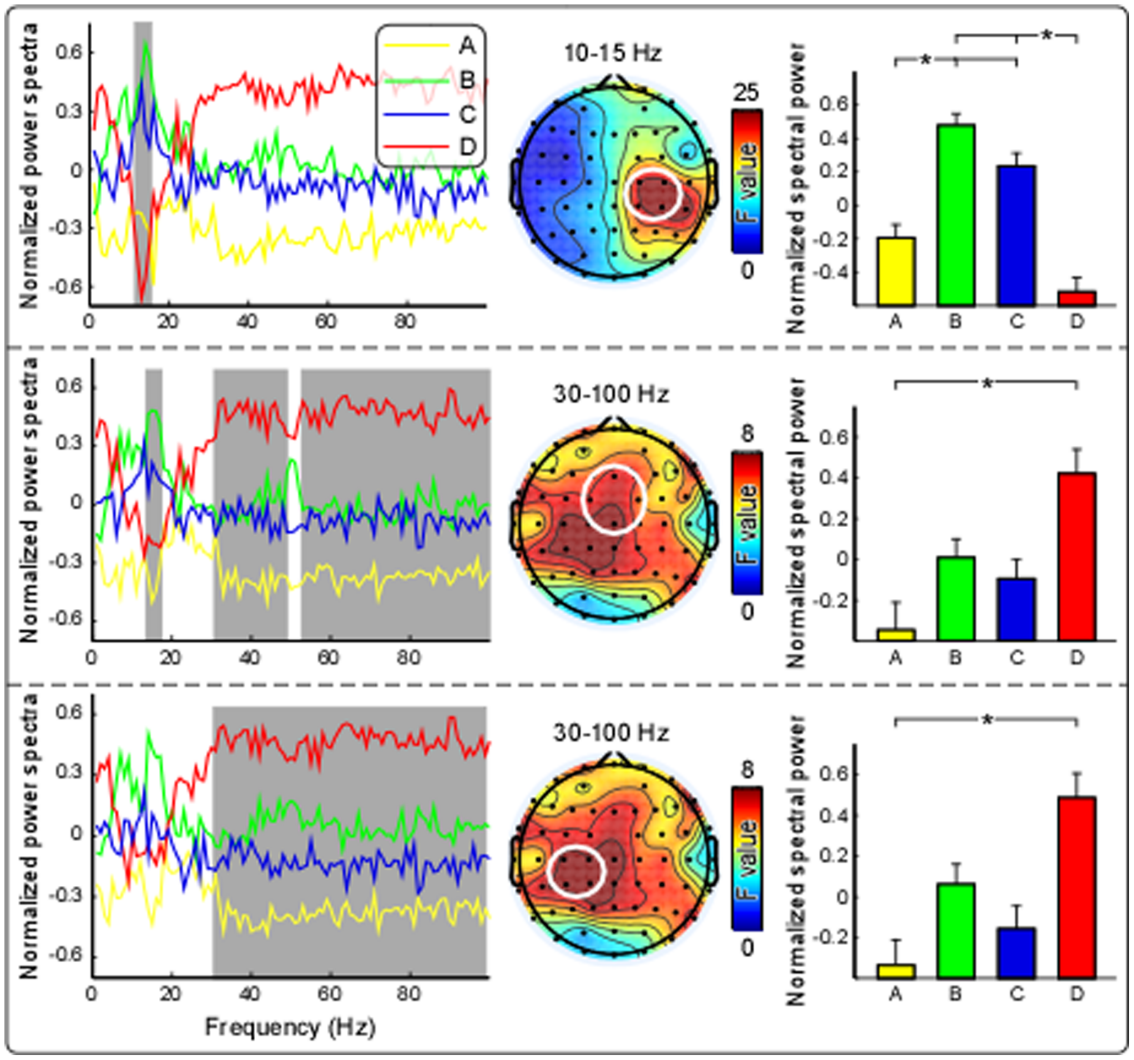
Comparison of normalized power spectra among four stimulation conditions. Normalized power spectra, measured at contralateral-central electrodes (C2, C4, CP2, and CP4; top), frontal-central electrodes (Fz, FC1, FC2, and Cz; middle), and ipsilateral-central electrodes (C1, C3, CP1, and CP3; bottom), were respectively displayed in yellow, green, blue, and red for conditions A, B, C, and D. As marked in grey, significant differences of power spectra across stimulation conditions were dominantly observed at contralateral-central electrodes from 10 to 15 Hz (top), at frontal-central electrodes from 30 to 55 Hz and from 60 to 100 Hz (middle), and at ipsilateral-central electrodes from 30 to 100 Hz (bottom). The summarized spectral power, measured at contralateral-central electrodes (top) within alpha band (10–15 Hz, top), at frontal-central (middle) and ipsilateral-central (bottom) electrodes within gamma band (30–100 Hz), were respectively marked in yellow, green, blue, and red, and were compared among four stimulation conditions. Error bars represent, for each condition, ± SEM across subjects. Asterisk * indicates a significant difference (P<0.05, Tukey’s post hoc tests).

Fig. 2 displays the grand average normalized power spectra of four stimulation conditions, which were respectively measured at contralateral-central electrodes (C2, C4, CP2, and CP4), frontal-central electrodes (Fz, FC1, FC2, and Cz), and ipsilateral-central electrodes (C1, C3, CP1, and CP3). As revealed by point-to-point repeated measures ANOVA, significant differences of power spectra across stimulation conditions were dominantly observed at contralateral-central electrodes (C2, C4, CP2, and CP4) within 10–15 Hz (i.e., upper alpha frequency band) (Fig. 2, top panel), at frontal-central (Fz, FC1, FC2, and Cz; 30–55 Hz and 60–100 Hz) and ipsilateral-central (C1, C3, CP1, and CP3; 30–100 Hz) electrodes within 30–100 Hz (i.e., gamma frequency band) (Fig. 2, middle and bottom panels, respectively).

The summarized spectral power within the alpha frequency band (10–15 Hz) at contralateral-central electrodes (C2, C4, CP2, and CP4), and within the gamma band (30–100 Hz) at frontal-central (Fz, FC1, FC2, and Cz) and ipsilateral-central (C1, C3, CP1, and CP3) electrodes were displayed in Fig. 2. As revealed by 4-level one-way repeated-measures ANOVA, the summarized spectral power at contralateral-central electrodes within the alpha frequency band differed significantly among four conditions (F (3, 111) = 19.84, P<0.001, partial Eta squared = 0.35) (Fig. 2). Post hoc analysis revealed that alpha power at contralateral-central electrodes were significantly lower in conditions A and D than those in conditions B and C (A vs. B: P = 0.003; A vs. C: P<0.001; D vs. B: P<0.001; D vs. C: P<0.001). In addition, at both frontal-central and ipsilateral-central electrodes, spectral power within the gamma frequency band also differed significantly among four conditions (frontal-central: F (3, 111) = 5.54, P = 0.001, partial Eta squared = 0.13; ipsilateral-central: F (3, 111) = 5.72, P = 0.001, partial Eta squared = 0.16) (Fig. 2). Post hoc analysis revealed that gamma power at frontal-central and ipsilateral-central electrodes were significantly higher in condition D than those in condition A (P = 0.006 and P = 0.003 respectively).

**Figure 3:**
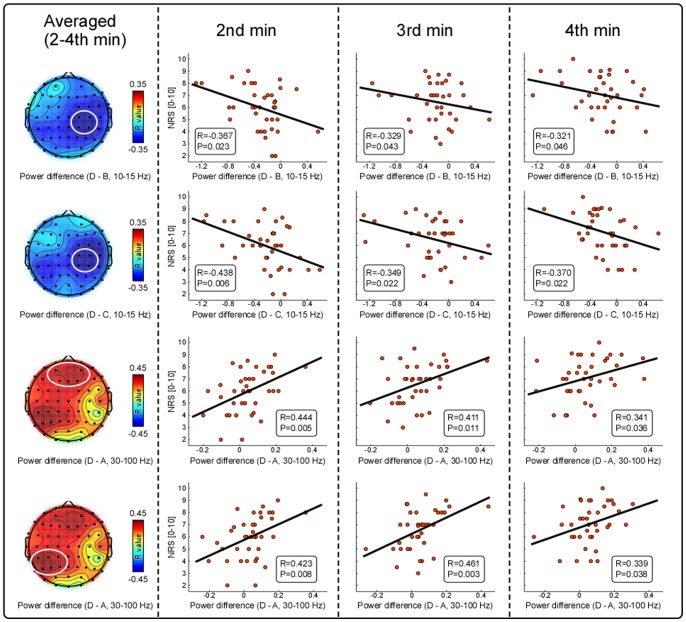
Relationships between spectral power differences and subjective intensity of pain perception. Negative correlations between spectral power differences (left panel, D–B, D–C) within alpha frequency band (10–15 Hz) and averaged subjective pain intensity during the interval of 2^nd^ to 4^th^ min were maximal at contralateral-central electrodes (C2, C4, CP2, and CP4). Positive correlations between spectral power difference (left panel, D–A) within gamma frequency band (30–100 Hz) and averaged subjective pain intensity during the interval of 2^nd^ and 4^th^ min were maximal at prefrontal-central (left: AF3, AF4, F1, Fz, and F2) and ipsilateral-posterior (right: CP1, CP3, CP5, P1, P3, and P5) electrodes. Specifically, the subjective pain intensity at each min were negatively correlated with alpha spectral power difference (D–B, D–C) at contralateral central electrodes (marked in the white circles), and positively correlated with gamma spectral power difference (D–A) at prefrontal-central and ipsilateral-posterior electrodes (marked in white circles). Each dot represents values from each subject, and black lines represent the best linear fit.

Linear correlation analysis revealed that negative correlations between spectral power differences (D–B and D–C) within the alpha frequency band (10–15 Hz) and averaged subjective pain intensity during the interval from 2^nd^ to 4^th^ min were observed at contralateral-central (C2, C4, CP2, and CP4) electrodes (left panel of Fig. 3), and positive correlations between spectral power differences (D–A) within the gamma frequency band (30–100 Hz) and averaged subjective pain intensity during the interval from 2^nd^ to 4^th^ min were observed at prefrontal-central (AF3, AF4, F1, Fz, and F2) and ipsilateral-posterior (CP1, CP3, CP5, P1, P3, and P5) electrodes (left panel of Fig. 3). Specifically, subjective pain intensity of each minute, was negatively correlated with alpha spectral power difference (D–B and D–C) summarized over contralateral central electrodes (D–B: 2^nd^ min [R = −0.367, P = 0.023], 3^rd^ min [R = −0.329, P = 0.043], 4^th^ min [R = −0.321, P = 0.043]; D–C: 2^nd^ min [R = −0.438, P = 0.006], 3^rd^ min [R = −0.349, P = 0.022], 4^th^ min [R = −0.370, P = 0.022]), and was positively correlated with gamma spectral power difference (D–A) summarized over prefrontal central (2^nd^ min [R = 0.444, P = 0.005], 3^rd^ min [R = 0.411, P = 0.411], 4^th^ min [R = 0.341, P = 0.036]) and ipsilateral-posterior electrodes (2^nd^ min [R = 0.423, P = 0.008], 3^rd^ min [R = 0.463, P = 0.003], 4^th^ min [R = 0.340, P = 0.037]).

**Figure 4:**
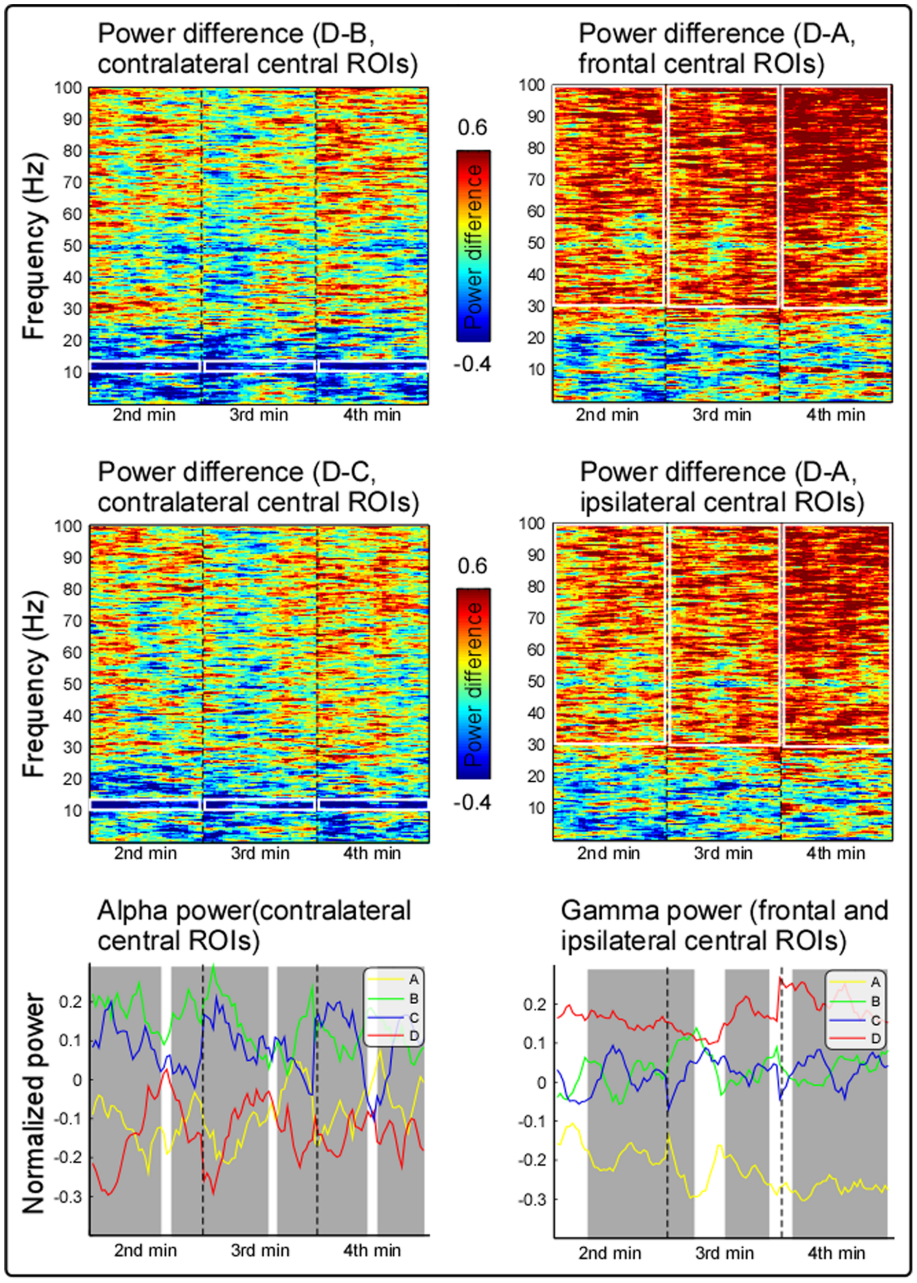
Time-varying normalized power spectra among four stimulation conditions. Grand averaged time frequency distributions of the spectral power density difference (left panel, D–B, D–C) were measured on contralateral central electrodes (C2, C4, CP2, and CP4), and power differences (right panel, D–A) were measured at frontal central (Fz, FC1, FC2, and Cz) and ipsilateral central (C1, C3, CP1, and CP3) electrodes. The dashed lines represent the start of each min, and the alpha suppression and gamma enhancement were marked in white rectangle on the time-frequency distribution of spectral power difference. Grand averaged time-varying spectral power curve at contralateral central electrodes within alpha (10–15 Hz) frequency band (left panel), and at frontal and ipsilateral central electrodes (right panel) within gamma (30–100 Hz) frequency band were also displayed in yellow, green, blue, and red for conditions A, B, C, and D. The intervals with significance difference across the conditions were marked in grey.

Fig. 4 displays the grand average time-varying normalized power spectra difference from 2^nd^ to 4^th^ minute with 10-s length of epochs, respectively calculated from spatial regions showing largest difference (contralateral-central electrodes [C2, C4, CP2, and CP4] for D–B and D–C; frontal-central [Fz, FC1, FC2, and Cz] and ipsilateral-central [C1, C3, CP1, and CP3] electrodes for D–A). It showed stable and persistent alpha suppression and gamma enhancement induced by long-lasting painful stimuli. As revealed by point-to-point repeated measures ANOVA, most time intervals of alpha spectral power curve over contralateral central electrodes were significantly different across conditions, while selective time intervals of gamma spectral power curve over frontal and ipsilateral central electrodes were significantly different.

**Figure 5:**
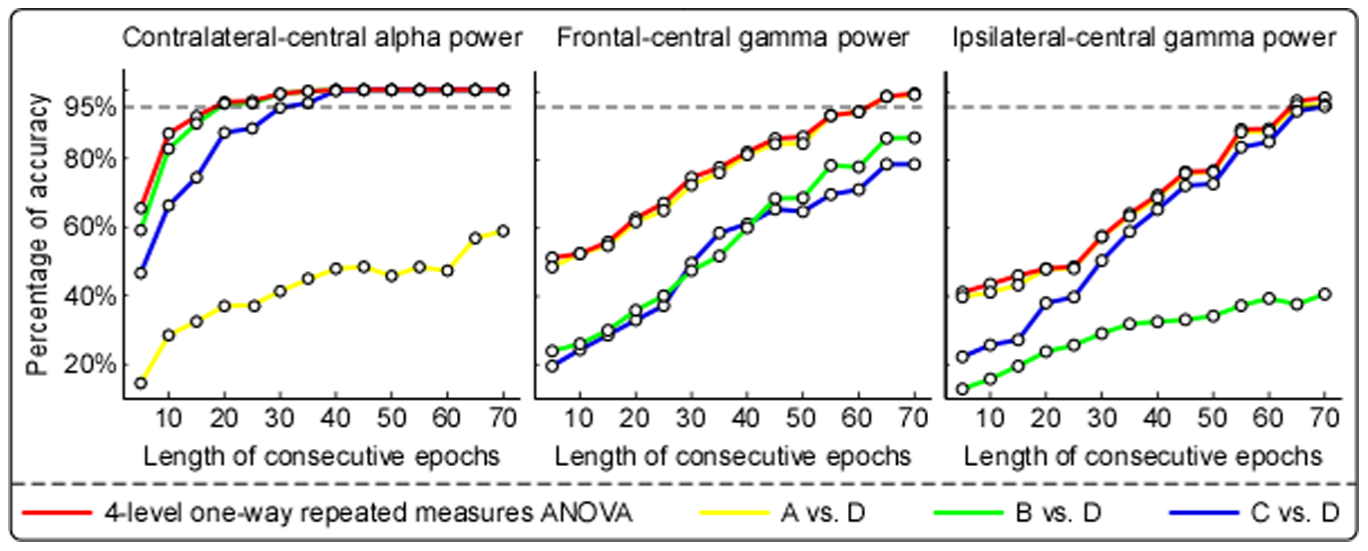
Percentage of accuracy to distinguish different tonic stimulation conditions using spectral power with different length of consecutive epochs. Each dot represents percentage of accuracy to distinguish different stimulation conditions in the corresponding length of consecutive epochs. Red lines represent the results to distinguish four stimulation conditions revealed by 4-level one-way repeated measures ANOVA. Lines in yellow, green, and blue respectively represent the results to distinguish conditions A and D, B and D, as well as C and D, which were revealed by post hoc analysis. The black dashed lines represent the percentage of accuracy at 95%. Spectral power within alpha frequency band (10–15 Hz) at contralateral-central electrodes (C2, C4, CP2, and CP4; left) could be used to distinguish conditions B and D, as well as C and D. Spectral power within gamma frequency band (30–100 Hz) at frontal-central (Fz, FC1, FC2, and Cz; middle) and ipsilateral-central electrodes (C1, C3, CP1, and CP3; right) could be used to distinguish conditions A and D.

Fig. 5 displays the percentage of accurately distinguishing different tonic stimulation conditions using spectral power with different length of epochs (ranging from 5 to 70 in step of 5) (1) within the alpha frequency band (10–15 Hz) at contralateral-central electrodes (C2, C4, CP2, and CP4), (2) within the gamma frequency band (30–100 Hz) at frontal-central (Fz, FC1, FC2, and Cz) and (3) ipsilateral-central (C1, C3, CP1, and CP3) electrodes. As revealed by 4-level one-way repeated measures ANOVA, the percentage of accurately distinguishing different stimulation conditions increased monotonically with the increase of the length of consecutive epochs. When using spectral power within the alpha frequency band at contralateral-central electrodes, at least 20 consecutive epochs (i.e., 20 s EEG recordings) were needed to achieve an accuracy of 95% for distinguishing different stimulation conditions. Post hoc analysis revealed that at least 20 and 30 consecutive epochs were needed to respectively distinguish conditions B and D, and conditions C and D, while it was impossible to distinguish conditions A and D even all 70 consecutive epochs were used. When using spectral power within gamma frequency band at both frontal-central and ipsilateral-central electrodes, at least 65 consecutive epochs (i.e., 65 s EEG recordings) were needed to achieve an accuracy of 95% for distinguishing different stimulation conditions. Post hoc analysis revealed that at least 65 consecutive epochs were needed to distinguish conditions A and D for both frontal-central and ipsilateral-central electrodes.

## Discussion

In this study, by comprehensively comparing EEG power spectra among the four experimental conditions, we obtained the following four main findings: (1) tonic pain induced oscillatory activities were characterized by stable and persistent suppression of alpha oscillations maximal over the contralateral-central region (conditions D vs. B and D vs. C), and enhancement of gamma oscillations over widespread cortical regions (conditions D vs. A); (2) alpha power at contralateral-central electrodes were significantly lower in conditions A and D than those in conditions B and C, and gamma power at both frontal-central and ipsilateral-central electrodes were significantly higher in condition D than those in condition A; (3) significant correlation between spectral power differences and subjective pain intensity were observed at contralateral-central electrodes within the alpha band (D–B and D–C), and at prefrontal-central and ipsilateral-posterior electrodes within the gamma band (D–A); (4) to achieve an accuracy of 95% for distinguishing different stimulation conditions, at least 20 s EEG recordings were needed if alpha spectral power over contralateral-central electrodes was used for the distinguishment, while 65 s EEG recordings were needed if gamma spectral power over frontal-central or ipsilateral-central electrodes was used for the distinguishment. All these findings indicated that the observed alpha oscillation suppression and gamma oscillation enhancement in condition D were both closely related to tonic pain perception, but they may reflect different aspects of the multidimensional experience of pain. The alpha response, easily influenced by attention, may primarily relate to top-down cognitive process, while the gamma response, more robust to attention but still significantly correlated with pain perception, may partly reflect tonic pain processing, relating to the interface between bottom-up stimulus-related process and top-down subject-driven cognitive process.

### Changes of Spontaneous Oscillatory Activities in Association with Tonic Heat Pain

Transient painful stimuli have been shown to suppress alpha rhythms mainly located in the sensorimotor and occipital cortices [2], [3], [5], [7]. This global suppression was thought to reflect the alerting function of pain, which opens the gates of sensory and motor systems and prepares the individual to process and react to stimuli of existential relevance [5]. Importantly, the laser induced alpha desynchronization that was reported to be correlated with subjective pain intensity [9] also increased with higher stimulus strength [2], indicating that pain induced alpha suppression reflects the central processing of painful stimulus. Along the same lines, gamma oscillations were also thought to play an important role in pain perception and processing, as it has shown that selective nociceptive stimuli could induce gamma oscillations over primary somatosensory cortex, whose amplitudes also vary with objective stimulus intensity and subjective pain intensity [6]. Meanwhile, an enhancement in gamma oscillations could also predict the subjective pain intensity regardless of stimulus repetition when delivering trains of three nociceptive stimuli and using different energies to elicit graded pain intensities [8], further suggesting the close relationship between gamma band oscillations and cortical activity subserving pain perception.

Here, to explore neural mechanisms related to persistent pain experience over a period of time, the recorded EEG data and subjective pain intensity ratings in the first and last minutes were discarded to rule out possible brain responses related to the sudden change of stimulation (i.e., the onset and offset of the stimulation). Similar with cortical response to transient painful stimuli, tonic heat pain also induced suppression of alpha oscillations over the contralateral-central region and enhancement of gamma oscillations over widespread cortical regions (Fig. 2), which were shown to be stable and persistent from the 2^nd^ to 4^th^ min (Fig. 4). Specifically, in condition D, the significantly decreased alpha oscillations and increased gamma oscillations were both significantly correlated with subjective pain intensity for each minute (Fig. 3), demonstrating such alpha suppression and gamma enhancement were always covarying with subjective pain intensity throughout tonic heat pain perception. These results in the present study indicated that the persistent and robust alpha oscillation suppression and gamma oscillation enhancement induced by long-lasting tonic painful stimuli, were closely associated with tonic heat painful stimulus perception.

Based on the close association between alpha oscillatory activity and cortical excitability [1], [5], [40], the observed significant suppression of alpha oscillations at contralateral-central electrodes revealed an increase of excitability in the sensorimotor cortex with the application of tonic heat painful stimuli. Meanwhile, the observed significant correlation between gamma spectral power difference and subjective pain intensity at prefrontal and posterior parietal regions, was quite consistent with previous evidence to show that prefrontal and parietal gamma band oscillations could reflect subjective perceptual experience [8], [41], [42], [43]. Such tonic pain induced enhancement of gamma oscillations probably related to the cortical representation of tonic painful stimulus processing, as enhanced gamma oscillations were interpreted as reflection of the bottom-up activation of cortical networks generating a subjective percept [44], [45]. It should also be noted that the observed broadly distributed gamma oscillations may reflect synchronization between cortical areas (frontal-central and ipsilateral-central regions) involved in tonic painful stimulus processing [41], [42], [43], [46] that should be further investigated in the future studies. Considering that pain is a unique experience that disrupts ongoing behavior, demands attention and urges the individual to react [5], [47], this global tonic heat pain induced change in cortical function and excitability may relate to the unique biological significance of pain, e.g., expecting to receive enhanced processing in relevant brain regions.

The signal-to-noise ratio of brain responses elicited by tonic stimulation is markedly lower compared with transient stimulation (i.e., the onset or offset of stimulus), thus tonic pain induced EEG response would not be reliable with short-interval EEG data. Since we have identified that the observed alpha suppression over contralateral central electrodes and gamma enhancement over frontal and ipsilateral central electrodes were closely related to tonic pain perception (Figs. 2–4), we tried to identify the minimal EEG recording interval that would be sufficient enough to distinguish different tonic stimulus conditions by measuring these tonic pain related oscillatory activities. Again, it is confirmed that measuring contralteral-central alpha oscillatory activities would be quite effective for distinguishing D vs. B or D vs. C (Fig. 5), while measuring frontal-central and ipsilateral-central gamma oscillatory activities would be effective for D vs. A (Fig. 5). The identified minimal recording interval for distinguishing different tonic stimulus conditions would provide important and instructive knowledge for pain clinics to classify the state of patients through continuous EEG.

### Effects of Attention on Tonic Heat Pain Related Response

It has been well accepted that the highly subjective and behaviorally relevant experience of pain is particularly susceptible to attentional modulations, which has been widely applied in both basic and clinical studies [21], [22], [23]. Functional imaging studies also showed that distraction from pain reduces pain-related activations in most brain areas that are related to sensory, cognitive and affective aspects of pain (e.g., primary and secondary somatosensory cortex, anterior cingulate cortex, thalamus, and insula) [22], [23], [48], [49], [50]. Specifically, Ohara [3] showed that attention to painful stimuli leads to enhanced alpha suppression over contralateral SI, and a higher perceived intensity was associated with greater and more widespread alpha decrease, indicating that pain induced alpha suppression could be greatly modulated by attention. Even more, by applying an oddball paradigm where the subject’s task was to count rare painful electrical stimuli applied to one finger, while ignoring frequent stimuli on a different finger, Hauk et al. [11] found that directed attention to pain was associated with stronger gamma oscillation enhancement in the contralateral sensorimotor areas. The effect of attention on alpha and gamma oscillation activities may reflect attention augmentation of processing that could enhance saliency of sensory signals and lead to preferential routing of the respective information transformation [11], [51], [52].

Consistent with previous studies reporting that a higher perceived intensity was associated with greater alpha oscillation suppression [2], [9], [17], significant negative correlations between spectral power difference (D–C, D–B) and subjective pain intensity were observed over contralateral-central electrodes (Figs. 2&3). In contrast with alpha band, we only observed difference of gamma oscillations between conditions D and A, but not between conditions D and C, indicating that the observed gamma oscillation enhancement (D vs. A) that was also significantly correlated with subjective pain intensity, is more robust to attention and reflects more about tonic painful stimulus processing rather than attention modulation. With a large body of evidences showing that directing attention to a location in sensory space could also induce a decrease of alpha activity in the cortical area representing that sensory space even when a stimulus is not presented [9], [24], [25], [53], [54] that has also been confirmed by regional cerebral blood flow studies [55], [56], tonic heat pain induced alpha oscillation suppression may largely result from the attention shift to the somatosensory stimuli on the left hand instead of directly reflecting the stimulus-related processing. The attention modulations on tonic pain induced alpha suppression further support the involvement of alpha oscillations in the mechanisms of top-down modulation, attention, and consciousness [57]. However, as previous studies have shown that gamma band activity enhanced during attentional selection of sensory information [27], [43], [58] and the subjects were required to focus attention on stimulus and rate subjective pain intensity in condition D during our experiment, we could not rule out the influence of high-level cognitive process on gamma oscillations.

As the modulation of attention affected alpha and gamma oscillatory activities in a different manner, we could hypothesize that attention modulation would affect alpha oscillation activities more than gamma oscillation activities. Our data showed that directing attention towards the long-lasting pain stimulus would significantly modulate alpha oscillatory activity over contralateral-central electrodes, but would not significantly modulate gamma oscillatory activity. This could even be supported by the proposal that oscillatory activity in the low frequency band would reflect more about top-down processing, while oscillations in high frequency bands would be more related to stimulus-dependent bottom-up processing [59]. Our data could thus fit with the idea that bottom-up processes show up more at higher frequencies than top-down processes.

When comparing the gamma activities between conditions D and B, no significant difference was observed at frontal-central or ipsilateral-central electrodes. It could be due to the following two reasons: (1) the signal-to-noise ratio of gamma oscillation is poor [14], [42], [43], since gamma oscillations are normally and easily contaminated by a lot of non-neural artifacts (e.g., cranial and ocular muscle activity) [60]; (2) gamma oscillations, in nature, capture multiple functions [6], [11], [42], [44], [61], [62], [63]. In other word, the enhancement of gamma oscillation activities could be observed in various experimental conditions (e.g., being elicited by sensory stimuli of various modalities, including visual, auditory, and somatosensory) [6], [8], [41], [44], [61], [64], [65]. Even in condition B (innoxious stimulus, 36°C), it would be reasonable to observe the enhancement of gamma oscillations (even not significant). Thus, even gamma oscillations are not specific to tonic pain perception, the observed enhancement of gamma oscillations (conditions D vs. A) that was significantly correlated with subjective pain intensity in the present study, at least would be partly due to tonic pain processing.

In summary, the stable and persistent alpha suppression over contralateral-central region and widespread gamma enhancement were both closely related to tonic heat pain perception, which may reflect different aspects of the multidimensional experience of pain. The alpha suppression in response to tonic heat pain primarily reflects high-level cognitive process, while the enhancement of gamma oscillation, partly reflects tonic pain processing, representing the summary functions of stimulus-driven process and top-down determinants of pain perception. Our current findings extend prior research regarding cortical mechanisms underlying the processing of extended noxious stimulation, and may have important implications for objectively and straightforwardly assessing pain responsiveness in pain research and clinical pain management.
